# Advanced Analytics Show Domain‐Specific Performance Changes After Anterior Cruciate Ligament Reconstruction in National Football League Players

**DOI:** 10.1002/ars2.70117

**Published:** 2026-10-28

**Authors:** Emmitt Hayes, Zoe Kerhoulas, Bogdan Matache, Michael Pickell

**Affiliations:** ^1^ Division of Orthopaedic Surgery The University of Ottawa Ottawa Ontario Canada

## Abstract

**Purpose:**

To evaluate return to play, performance, and assess performance changes after anterior cruciate ligament reconstruction using advanced statistics in National Football League players.

**Methods:**

National Football League players who underwent anterior cruciate ligament reconstruction (ACLR) between 2017 and 2024 were identified using publicly available databases and injury reports. Demographic and outcome data for 1 year preinjury to 2 years postinjury were obtained. Return to play was defined as being listed as active for a regular season game after injury. Return to performance was defined as returning to greater than 90% of preinjury performance. Our primary performance outcome was Sports Information Solutions points above replacement (PAR). Binary logistic regression was used to determine factors associated with return to play and return to performance. Preinjury and postinjury performance data at the first and second postinjury year were compared with paired *t*‐tests.

**Results:**

We included 237 players in return‐to‐play and 173 players in return‐to‐performance analyses. Of players, 75.5% returned to play; players with more preinjury games played, more index games played, starters, and players with greater preinjury PAR were more likely to return to play (*P* = .03, *P* < .001, *P* < .001, *P* < .001, respectively). Fifty‐one percent of players who returned to play returned to performance; younger age and players with lower preinjury PAR were significantly more likely to return to performance (*P* = .004, *P* < .001). Total PAR was significantly decreased at the postinjury year 1 and year 2 seasons (*P* = .002, *P* = .004), driven at both time points by significantly decreased rushing (*P* = .02, *P *= .001) and pass coverage (*P* = .003, *P* = .03) PAR.

**Conclusions:**

After anterior cruciate ligament reconstruction, National Football League players with more preinjury games played and higher preinjury performance are more likely to return to play; younger players with lower preinjury performance are more likely to return to performance; and there is a significant decrease in overall performance 1 and 2 years after injury, driven by decreased rushing and pass coverage performance.

**Level of Evidence:**

Level IV, retrospective case series.

## Graphical abstract

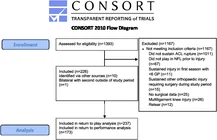

Anterior cruciate ligament (ACL) ruptures are among the most common musculoskeletal injuries sustained by National Football League (NFL) players, with a per‐season injury risk of 2% per player.^1^ ACL injuries can have devastating effects on NFL players, with up to 44% of players failing to return to play after injury.^2^ Comparisons of pre‐ and postinjury performance have showed declines in games played (GP), games started, snap count, approximate value,^2^ interceptions per game,^3^ targets, receptions, receiving yards, touchdowns,^4^ and power rating.^5^

The use of analytics for evaluating player performance has exploded in the NFL, with teams employing both internal staff and partnering with private analytic firms to improve player evaluation and in‐game decision‐making. Analytics are now used when evaluating potential draft choices, trade targets, and when deciding which players to start. Furthermore, teams use advanced analytics to guide play calling and in‐game decision‐making. Despite the implications of ACL injuries in NFL players, return‐to‐performance outcomes using advanced statistics have not been evaluated in NFL football players. Prior studies have relied primarily on traditional measures, including return to play, GP, and other traditional metrics of player performance.^2^, ^3^, ^4^, ^5^, ^6^, ^7^, ^8^ Although return to sport and GP are important, the primary goal of injured athletes and their coaches is to both return to the prior performance level and maximize their future performance.^9^ Traditional metrics of NFL performance such as rushing or receiving yards, receptions, touchdowns, tackles, or fumble recoveries are highly dependent on a player's situation and volume of usage and are not directly related to team performance or wins. Although some studies have used approximate value as a performance measure, this is a combination of traditional metrics and thus shares the same limitations. Although return to performance after orthopaedic procedures has been assessed in the National Hockey League, Major League Baseball, and National Basketball Association players using advanced statistics, advanced statistics have not been used to assess outcomes after orthopaedic procedures in NFL players.^10^, ^11^, ^12^

Advanced statistics in professional football are based on expected points added (EPA), a metric that uses historical play‐by‐play data to estimate either the number of points scored or the win probability for teams in similar situations.^13^ Expected points or win probability is then compared before and after each play to calculate the change in expected value. Each player's performance is measured against the historical average at the same position in comparable situations to assign that player a share of the EPA.^13^ These methods allow for comparisons within and between positions, evaluation of every player on every play, and a direct assessment of each player's contribution to points scored and overall wins.

The purpose of this study was to evaluate return to play, performance, and assess performance changes after ACL reconstruction using advanced statistics in NFL players. We hypothesized that approximately half of players who returned to play will return to the same performance level as preinjury.

## METHODS

### Study Design

Institutional Review Board approval was not required for this study because publicly available data were used. We identified NFL players who underwent ACL reconstruction between the 2017 and 2024 NFL seasons using the Pro Sports Transactions Archive—a database of all NFL transactions including movement to and from injured reserve and the Physically Unable to Perform list, which has been used extensively in prior epidemiological research.^14^, ^15^, ^16^, ^17^, ^18^ We identified players in the database who were listed as sustaining knee, knee ligament (ACL, posterior cruciate ligament, posterolateral corner, posteromedial corner), meniscal, chondral, or patellofemoral injuries. News articles, press releases, injury reports, player profiles, and biographies were reviewed for each player identified via database searching to determine whether each player sustained an ACL rupture and underwent surgical reconstruction. We searched the database in the same fashion for all eligible players to determine whether they sustained other orthopaedic injuries requiring surgical management during the study period.

We included players who sustained a native ACL rupture, underwent surgical ACL reconstruction, and had played at least 1 NFL season prior to injury. In cases where a player sustained an ACL rupture in their first NFL season, we included players who had played at least 6 games and used the injury season as the index season. In all other players, the first full preinjury season was used as the index season. We excluded players who did not undergo ACL reconstruction, players undergoing multiligament knee reconstruction, players who sustained a repeat rupture of a previously reconstructed ACL, players who sustained a repeat rupture or contralateral ACL rupture within the study period, players who had complications requiring revision surgery within the study period, players who sustained another orthopaedic injury requiring surgical management within the study period, and players who did not have media reports confirming that they underwent surgical ACL reconstruction.

### Data Collection

Birthday, height, weight, draft year, career duration, pro bowl status, and total career GP were obtained from Pro Football Reference and abstracted in a spreadsheet. Outcome and position data were obtained from Sports Information Solutions. Sports Information Solutions is a private analytics company that works with 11 NFL, 21 Major League Baseball, and 7 National Basketball Association teams to provide both injury and performance data, which are used for game planning and player evaluation as well as with the Major League Baseball Players Association, Entertainment and Sports Programming Network, and multiple major sportsbooks. Our primary performance outcome measure was overall Sports Information Solutions points above replacement (PAR) (which is the summation of PAR achieved in each performance domain of passing, rushing, receiving, blocking, pass coverage, pass rush, and run defense). Secondary performance outcomes were Sports Information Solutions PAR metrics for all specific performance domains.

Sports Information Solutions has quantified and released statistics since the 2016 NFL season for all NFL players active in at least 1 regular season offensive or defensive snap using their Total Points Framework. This is an expected points framework similar in nature to previously described EPA frameworks.^13^ Under an EPA framework, the number of points scored at the end of a drive by historical teams in similar situations is compared before and after a play to determine the number of points added or subtracted from a team's expected scoring total in each play.^13^ To evaluate a single player, their performance on each play is compared with the historical average performance at the same position in similar situations to determine the number of points that a player's performance on a given play either adds or subtracts from the team's total.^13^ This can then be expressed relative to a replacement‐level player at their position in each specific performance domain (passing, rushing, receiving, blocking, pass coverage, pass rush, and run defense) and is termed PAR. PAR is advantageous because it allows for comparison both within and between positions, evaluates every player on every play regardless of whether they were directly involved, and provides a direct assessment of performance that allows for the evaluation of every player's contribution to points scored and wins.^13^ Return to play was defined as being listed as active for a regular season game after injury. Return to performance was defined as returning to greater than 90% of preinjury performance.

To generate the Sports Information Solutions PAR score for each player, each offensive and defensive play in every season is analyzed. The total EPA (or subtracted) in each play are assigned to players based on a predetermined metric using each player's individual contribution to the play compared with historical performance at each position. This is based on situation, formation, play type, and formation of the opposition. The specific domain that the player was participating in (i.e., passing, rushing, receiving, blocking, pass coverage, pass rush, and run defense) is recorded, and the PAR for this specific domain is recorded; a player's overall PAR is the summation of the PAR added in each domain. By evaluating players in this fashion, each player can be assessed on each play regardless of whether they were directly involved. For example, a receiver who runs an excellent route and gets open adds expected points to a passing play, even if poor offensive line (OL) play allows a sack before the quarterback (QB) has a chance to throw the ball. Likewise, the poor OL play will be quantified as losing points in that particular play, with the specific OL player(s) whose performance deviation allowed the sack credited with losing points for their team. Thus, both good and bad plays by different players on a given play are recognized and recorded, generating a statistic that isolates the individual performance for every play the player is on the field. This statistic is then adjusted for opponent performance to further isolate an individual player's performance and can be expressed relative to a replacement player, defined as rostered players who are not involved in the first 15 offensive or defensive plays of the game. Many teams script the first 15 plays on offense and defense, so players not involved in these plays are likely seen by a team as replaceable.^19^ We selected PAR as our primary outcome metric because we feel that this metric most appropriately assesses player value; replacement‐level players can be easily replaced at a given position and thus are more appropriate than comparison to an average player. A full description of Sports Information Solutions methodology is available online.^19^, ^20^

### Power Calculation

We included all eligible players who sustained an ACL injury 1 or more seasons after Sports Information Solutions began collecting PAR data. As such, ex‐ante sample size calculation was performed. Using the mean (6.3) and standard deviation (11.9) preinjury PAR among cases, 63 players were needed to detect a one‐half standard deviation performance decline at an alpha of 0.05 and 80% power.

### Statistical Analysis

Data analysis was performed using IBM SPSS Statistics version 29. The index season was defined as the first full season prior to injury. Players injured in their first season who played more than one‐third of the season were included with the injury season defined as the index season. Binary logistic regression was used to assess factors associated with return to play and return to performance. Overall and domain‐specific performance in the index season was compared with the first and second postinjury seasons using paired *t*‐tests. *P* values for the primary outcomes (total PAR in postinjury year 1 and year 2) were Bonferroni corrected for multiple comparisons; however, our secondary analysis of domain‐specific changes was not Bonferroni adjusted given that this secondary analysis was exploratory in nature and we thus wanted to maximize the statistical power of this analysis.^21^, ^22^ Bonferroni‐adjusted *P* values were reported. In all cases, *P* < .05 was defined as a significant result.

## RESULTS

We included 237 total players (9 QB, 19 running back, 17 tight end, 36 wide receiver (WR), 35 OL, 48 defensive back, 41 linebacker (LB), 28 defensive line, and 4 special teams) who sustained ACL ruptures at a mean age of 26.0 (standard deviation 2.7) after a mean of 3.8 (standard deviation 2.5) preinjury seasons. One hundred included players were starters. Players played a mean of 2.2 (standard deviation 2.0) postinjury seasons and had a mean career duration of 6.0 (standard deviation 3.1) seasons with an average of 70.9 (standard deviation 43.8) career GP. Mean height was 187.2 (standard deviation 7.2) cm, mean weight was 108.4 (standard deviation 20.0) kg, and mean body mass index was 30.7 (standard deviation 4.1). Figure 1 displays a flow diagram of included players; 1393 total players with knee injuries were assessed for eligibility. A total of 237 eligible players had played in the NFL prior to injury and were included in return‐to‐play analysis, 173 returned to play and were included in return‐to‐performance analysis, and 169 had preinjury and postinjury PAR data and were included in our comparison of pre‐ and postinjury performance.

**FIGURE 1:**
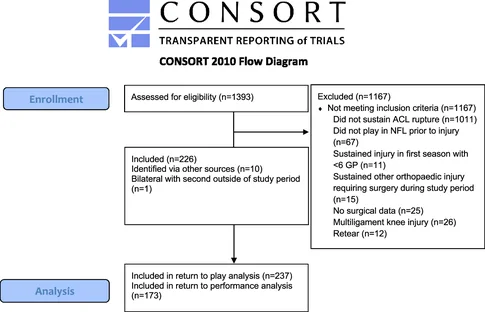
CONSORT diagram showing the flow of patients through this study. (ACL, anterior cruciate ligament; CONSORT, Consolidated Standards of Reporting Trials; GP, Games Played; NFL, National Football League.)

### Return to Play

Two hundred thirty‐seven players were included in return‐to‐play analysis. Of these, 179 (75.5%) returned to play in at least 1 NFL game. Return‐to‐play rates by position were 8/9 for QB, 13/19 for running back, 26/36 for WR, 14/17 for tight end, 27/35 for OL, 37/48 for OL, 28/41 for LB, 22/28 for LB, and 4/4 for special teams. Table 1 displays results of univariate logistic regression; notably, players with more preinjury GP, more GP in the index season, starters, and players with greater performance in the preinjury season were significantly more likely to return to play.

**TABLE 1:** Factors Associated With Return to Play

	Odds Ratio	95% CI Lower Bound	95% CI Upper Bound	*P* Value
Age at injury	0.94	0.85	1.05	.27
BMI	1.02	0.95	1.10	.60
Preinjury seasons	1.07	0.95	1.22	.27
Preinjury GP	1.01	1.00	1.02	.03
Index GP	1.19	1.11	1.26	<.001
Starter	5.63	2.61	12.15	<.001
Index PAR	1.13	1.06	1.21	<.001
Position (relative to WR)				.86
DB	1.29	0.48	3.49	.61
DL	1.41	0.44	4.50	.56
LB	0.83	0.31	2.21	.71
OL	1.30	0.44	3.80	.63
QB	3.08	0.34	27.86	.32
RB	0.83	0.25	2.80	.77
TE	1.80	0.42	7.61	.43

*Note*: Odds ratios, 95% confidence intervals, and *P* values for univariate logistic regression assessing factors associated with return to play.

BMI, body mass index; CI, confidence interval; DB, defensive back; DL, defensive line; GP, games played; LB, linebacker; OL, offensive line; PAR, points above replacement; QB, quarterback; RB, running back; TE, tight end; WR, wide receiver.

### Return to Performance

One hundred seventy‐three players returned to play and had postinjury performance data and were thus included in return‐to‐performance analysis. About 89/173 players who returned to play returned to performance by their second season after ACL injury. Return‐to‐performance rates by position were 4/8 for QB, 4/13 for running back, 18/26 for WR, 10/14 for tight end, 10/27 for OL, 19/36 for defensive back, 10/25 for LB, 14/22 for defensive line, and 0/2 for special teams. Table 2 displays results of univariate logistic regression; notably, younger players and players with lower levels of preinjury performance were significantly more likely to return to their preinjury performance level.

**TABLE 2:** Factors Associated With Return to Performance

	Odds Ratio	95% CI Lower Bound	95% CI Upper Bound	*P* Value
Age at injury	0.84	0.75	0.95	.004
BMI	0.95	0.89	1.03	.21
Preinjury seasons	0.89	0.78	1.00	.06
Preinjury GP	0.99	0.98	1.00	.06
Index GP	0.96	0.89	1.03	.25
Starter	0.58	0.32	1.07	.08
Index PAR	0.89	0.84	0.93	<.001
Position (relative to WR)				.10
DB	0.50	0.17	1.43	.20
DL	0.78	0.23	2.59	.68
LB	0.30	0.09	0.94	.04
OL	0.26	0.08	0.82	.02
QB	0.44	0.09	2.24	.33
RB	0.20	0.05	0.84	.03
TE	1.11	0.27	4.63	.89

*Note*: Odds ratios, 95% confidence intervals, and *P* values for univariate logistic regression assessing factors associated with return to performance.

BMI, body mass index; CI, confidence interval; DB, defensive back; DL, defensive line; GP, games played; LB, linebacker; OL, offensive line; PAR, points above replacement; QB, quarterback; RB, running back; TE, tight end; WR, wide receiver.

### Comparison of Preinjury and Postinjury Performance

One hundred sixty‐nine included players had pre‐ and postinjury performance data and thus were included in performance analysis. Table 3 displays a comparison of pre‐ and postinjury performance. Notably, there was a significant decrease in overall performance 1 in 2 years after surgery. Analysis of specific performance domains showed a significant decrease in rushing and pass coverage performance 1 and 2 years after surgery and a significant decrease in blocking performance 1 year after surgery.

**TABLE 3:** Comparison of Pre‐ and Postinjury Performance

	Index Mean (95% CI)	I + 1 Mean (95% CI)	*P* Value	I + 2 Mean (95% CI)	*P* Value
Passing PAR (10, 7)	22.9 (7.6‐38.2)	13.4 ( − 3.2 to 30.0)	.34	37.1 (20.2‐54.0)	.62
Rushing PAR (30, 23)	4.9 (2.1‐7.7)	1.3 ( − 0.5 to 3.1)	.02	− 1.7 ( − 4.7 to 1.3)	.001
Receiving PAR (52, 32)	2.6 (0.9‐4.3)	1.9 (0.3‐3.5)	.54	3.0 (0.7‐5.3)	.77
Blocking PAR (55, 41)	2.2 (1.0‐3.4)	0.4 ( − 0.4 to 1.2)	.01	1.6 (0.4‐2.8)	.59
Pass coverage PAR (70, 47)	3.0 (1.4‐4.6)	0.1 ( − 0.8 to 1.0)	.003	1.1 ( − 0.2 to 2.4)	.03
Pass rush PAR (77, 54)	2.0 (0.7‐3.3)	0.9 ( − 0.1 to 1.9)	.07	1.8 (0.1‐3.5)	.49
Run defense PAR (82, 56)	0.7 (0.0‐1.4)	0.1 ( − 0.6 to 0.8)	.29	0.5 ( − 0.4 to 1.4)	.87
Total PAR (169, 114)	6.3 (4.5‐8.1)	2.2 (0.8‐3.6)	.002	5.1 (3.1‐7.1)	.004

*Note*: Comparison of preinjury and postinjury performance. Means and 95% confidence intervals were reported. Paired *t*‐tests used to compare both postinjury time points to the index season. n for the postinjury year 1 and 2 seasons listed respectively in brackets in the first column of each row.

CI, confidence interval; PAR, points above replacement.

## DISCUSSION

The most important findings of this study are that among NFL players who sustain an ACL rupture, players with more preinjury GP, starters, and players with greater performance levels are more likely to return to play. Players who are younger and have lower levels of preinjury performance are more likely to return to their preinjury performance level. Mean overall performance significantly declined both 1 and 2 years after surgery, which our analysis suggested was caused by a significant decline in rushing and pass coverage performance. Position did not significantly impact the likelihood of return to play or return to performance.

Our results suggest that there are domain‐specific performance changes after return to play in professional football players. Our analysis revealed a significant decline in rushing and pass coverage performance after ACL reconstruction. Effective rushing and pass coverage in football require the ability to cut and pivot at high speeds in response to the movements of both offensive and defensive players, which our results suggest may not fully return to baseline after ACL reconstruction. There was no significant change in passing, receiving, pass rush, or run defense performance, suggesting that players can effectively return to performance in these domains after ACL reconstruction.

It has been shown that NFL players have a 2% per year risk of sustaining an ACL rupture, with higher risk in the preseason.^1^ Multiple previous works have assessed return‐to‐play and return‐to‐performance changes in NFL players after ACL reconstruction using traditional statistics. Our 76% return‐to‐play rate after ACL reconstruction is higher than return‐to‐play rates in prior works of 56% to 63%;^2^, ^23^, ^24^ our rate is likely higher because we excluded players who did not play in a regular season NFL game prior to injury. When pooling performance changes among all players, prior work has showed a decrease in overall approximate value,^2^ which is consistent with our finding of a significant decrease in overall performance after ACL reconstruction. Prior work has showed no change in passing performance after return from ACL reconstruction,^4^, ^8^ which is consistent with our results. Some authors have found decreased rushing performance after ACL reconstruction in the domains of attempts, yards, and touchdowns;^4^ our results suggest a similar decrease in rushing performance. Some previous authors have found decreased performance among WRs after return from ACL reconstruction, specifically, in the domains of fantasy points,^4^ targets, receptions, and receiving yards;^6^ our differing results suggest that the previously observed performance decline may be because of changes in usage rather than changes in player ability. Prior studies assessing performance changes in offensive and defensive lineman have showed a 64% return‐to‐play rate and no change in GP or started.^7^ Defensive players returning from ACL reconstruction have been shown to play fewer games per year and make fewer interceptions and tackles per game,^3^ which is congruent with our results showing decreased pass coverage performance.

A large advantage of this study was the use of advanced statistics for performance assessment. Traditional metrics of performance evaluation in football players share significant limitations.^13^ Traditional NFL statistics can be collected only on the plays in which an individual play is targeted (which is a small fraction of the total plays in which a player participates) and fail to account for the context of a particular play on game outcome.^25^ For example, gaining 5 rushing yards on third down with 4 yards remaining has a much different impact on a team's chance of winning a game than gaining 5 rushing yards on third down and 7 yards remaining; one of these results in a lengthened drive, whereas the other results in a turnover. Using EPA‐based statistics for performance evaluation ensures that an individual player's performance is adjusted for teammate and opponent performance, adds context to a player's performance, increases the sample size on which a player is evaluated, and directly evaluates a player's contribution to a team's points scored and wins.^13^, ^25^ Furthermore, these metrics can be used to compare players at separate positions and allow for the assessment of players at positions that are not typically evaluated with traditional statistics (defensive players and offensive linemen).^13^

### Limitations

This study has several limitations. Although we achieved statistical significance in multiple domains, the clinical significance of these results has not yet been established in the literature. We included all seasons for which advanced performance data were collected; however, given that these data have only been available since 2016, this did limit our sample size as well as the analysis and inference we were able to draw when making comparisons among individual performance domains; although our study was adequately powered to detect a one‐half standard deviation decline in overall and defensive (pass coverage, pass rush, and run defense) performance, we were underpowered to detect a one‐half standard deviation performance decline in offensive performance domains (passing, rushing, receiving, and blocking performance). The use of publicly available data does have limitations because we did not have detailed injury‐ and procedure‐specific information, which limited our analysis, particularly with respect to concomitant injuries and concomitant surgical procedures such as concomitant meniscal work, which may impact return‐to‐play and return‐to‐performance rates.^26^ Furthermore, we did not have data on prior ipsilateral non‐ACL knee surgery, which may have influenced our results. Finally, our analysis was limited to the first 2 postinjury seasons and as such may have failed to account for postoperative changes that occur more remotely from surgery.

## CONCLUSIONS

After ACL reconstruction, NFL players with more preinjury games played and higher preinjury performance are more likely to return to play, younger players with lower preinjury performance are more likely to return to performance, and there is a significant decrease in overall performance 1 and 2 years after injury, driven by decreased rushing and pass coverage performance.

## DISCLOSURES

The authors (B.M., M.P.) declare the following financial interests/personal relationships which may be considered as potential competing interests: B.M. reports a relationship with Arthrex that includes funding grants and Pendopharm that includes funding grants. M.P. reports a relationship with CONMED Corporation that includes consulting or advisory and Arthrex that includes consulting or advisory. The other authors (E.H., Z.K.) declare that they have no known competing financial interests or personal relationships that could have appeared to influence the work reported in this article.
